# Insulin-Like Growth Factor Binding Protein-3 Binds to Histone 3

**DOI:** 10.3390/ijms22010407

**Published:** 2021-01-02

**Authors:** Apurva Bhardwaj, Kumar Alok Pathak, Anuraag Shrivastav, Shailly Varma Shrivastav

**Affiliations:** 1 Department of Biology, The University of Winnipeg, Winnipeg, MB R3B 2G3, Canada; bhardwaj.apurva20@gmail.com (A.B.); a.shrivastav@uwinnipeg.ca (A.S.); 2 Research Institute of Oncology and Hematology, CancerCare Manitoba, Winnipeg, MB R3E 0V9, Canada; apathak@cancercare.mb.ca; 3 Department of Surgery, University of Manitoba, Winnipeg, MB R3A 1R9, Canada; 4 VastCon Inc., Winnipeg, MB R3P 1J9, Canada

**Keywords:** IGFBP-3, histone 3, protein-protein interaction

## Abstract

Insulin-like growth factor (IGF) binding protein-3 (IGFBP-3) is an essential protein that regulates cellular processes such as cell proliferation, apoptosis, and differentiation. It is known to bind with several proteins to carry out various cellular functions. In this study, we report for the first time that IGFBP-3 is a histone 3 (H3) binding protein. Sub-cellular fractionation was performed to separate into cytosolic fraction, nucleic acid binding protein fraction and insoluble nuclear fraction. Using ligand blot analysis, we identified a ~15 kDa protein that can interact with IGFBP-3 in the insoluble nuclear fraction. The 15 kDa protein was confirmed as histone 3 by far-Western blot analysis and co-immunoprecipitation experiments. A dot-blot experiment further validated the binding of IGFBP-3 with H3. The intensity of IGFBP-3 on dot-blot showed a proportional increase with H3 concentrations between 2.33 pmol–37.42 pmol. Our results support the presence of protein-protein interaction between IGFBP-3 and H3. The physical binding between IGFBP-3 and H3 could indicate its yet another cellular role in regulating the chromatin remodeling for gene transcription.

## 1. Introduction

Insulin-like growth factor binding protein 3 (IGFBP-3) is an essential protein involved in regulating various cellular processes, including cell proliferation, apoptosis, cell survival and differentiation [1]. The delivery of insulin like growth factors (IGF) produced primarily by the liver cells to the target cells via the bloodstream is a well-established endocrine function of IGFBP-3. One of the most abundant of the six known IGFBPs, IGFBP-3 circulates in the bloodstream bound with acid-labile subunit (ALS) and IGFs in the form of a ternary complex. IGFBP-3 can function in an IGF-dependent as well in an IGF-independent manner. The IGF-dependent primary function of IGFBP-3 is to transport IGFs sequestered within the ternary complex to the IGF receptors (IGFRs) for the initiation of a cascade of downstream signaling events. On the contrary, the IGF-independent roles of IGFBP-3 are elicited by internalization through various endocytic mechanisms, and translocation into the nucleus to interact with intracellular proteins.

The C-terminal domain of IGFBP-3 possesses a nuclear localization signal (NLS) which plays an essential role in the translocation of IGFBP-3 into the nucleus upon binding with importin-β [2,3]. Through the IGF-independent mechanisms, IGFBP-3 serves as a gate-keeper protein leading to cell cycle arrest and apoptosis and as a caretaker protein it is involved in the regulation of DNA repair, induction of autophagy, and regulation of cell survival through the modulation of sphingolipids [4,5,6,7,8]. The antiproliferative effects of several molecules like retinoic acid [9,10,11], transforming growth factor-β (TGF-β) [12], tumor necrosis factor-α (TNF-α) [13], and vitamin D [14,15,16,17] is due to their ability to augment the levels of IGFBP-3 expression in cells. IGFBP-3 has been reported to bind with several proteins on the plasma membrane to carryout various IGF-independent functions like association with transmembrane protein 219 (TMEM219) or IGFBP-3R [18], caveolin-1, β 1 integrin receptor [19], epidermal growth factor receptor (EGFR) [6], transferrin/transferrin receptor [20,21], type V transforming growth factor-β or low density lipoprotein 1 α 2M receptor [22,23] and autocrine motility factor or phosphogluco isomerase [24]. Intracellular IGFBP-3 binding proteins include glucose regulated protein 78 (GRP78) [5,25], humanin [26], *N*-acetylgalactosaminyltransferase 14 [27], importin-β [2,3], RNA polymerase binding II subunit 3 (Rpb 3) [28], nuclear retinoid X receptor (RXR) [29], retinoid acid receptor (RAR) [2], Nur 77 [20], peroxisome proliferator activated receptor -gamma (PPAR-γ) [30], vitamin D receptor (VDR) [31], catalytic subunit of DNA protein kinase (DNA-PKcs) [6] that play an essential role in implementing the ligand-independent action of IGFBP-3.

In this study, using biochemical techniques, we report for the first time that IGFBP-3 can physically interact with histone 3, which is suggestive of its ability to interact with nucleosomes and chromatin.

## 2. Results

**Figure 1:**
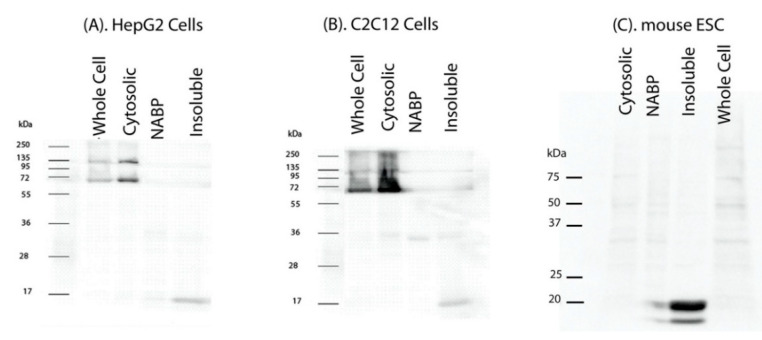
IGFBP-3 ligand blot with 15 kDa bands in the insoluble fraction. (**A**). HepG2, (**B**). C2C12 and (**C**). mouse embryonic stem cells (ESC) were cultured to 80% confluency, starved and lysed to fractionate cytosolic and nuclear fractions. The nuclear fraction was further sub-fractionated into nucleic acid binding protein (NABP) fraction and insoluble nuclear fraction. Whole cell lysates and the fractions prepared were resolved on a 12% SDS PAGE gel, transferred onto membrane. The blots were probed with biotinylated IGFBP-3. Band size corresponding to ~15 kDa was observed in all the cell lines tested.

IGFBP-3 protein localizes in the nucleus and is also known to bind to various proteins. Nuclear IGFBP-3 is known to play a role in gene transcription and DNA repair. Therefore, we were interested in investigating the binding partners of IGFBP-3 in the nucleus. We used HepG2 cells to study the interacting proteins of IGFBP-3. HepG2 cells were fractionated into cytosolic and nuclear fractions. The nuclear fraction was further sub-fractionated into nucleic acid-binding protein (NABP), containing the transcription factors, and insoluble nuclear fraction, containing nucleosomes. The purity of the fractions was characterized using tubulin, a marker for cytosolic fractions and histone 3, a marker for the insoluble fractions [32,33] (Figure S1). The isolated cell fractions and whole cell lysate were used to perform IGFBP-3 ligand bloting using biotinylated IGFBP-3. The proteins from various fractions were separated on a 12% SDS-PAGE and transferred onto nitrocellulose membrane for IGFBP-3 ligand blotting using biotinylated human IGFBP-3. The membranes were treated with streptavidin-HRP and the complex was visualized using chemiluminescent regent. There were characteristic bands indicative of the proteins bound with IGFBP-3 obtained in all the fractions, however, in this study we were interested in the identification of the ~15 kDa band obtained in the insoluble fraction (Figure 1A).

To further determine if this ~15 kDa IGFBP3 binding protein in the insoluble fraction was specific to liver cells, we also performed ligand blotting using C2C12 mouse myoblast cells and mouse embryonic stem cells (ESC). The cytoplasmic and nuclear fractions were isolated as was performed for the HepG2 cells. The cytosolic, NABP, insoluble nuclear fractions, and whole cell lysates of C2C12 and ESC were subjected to the ligand blot analysis. We observed a prominent band at ~15 kDa in the insoluble fraction of all the cell lines investigated (Figure 1B,C), suggesting that IGFBP-3 binds to a protein with a molecular weight of ~15 kDa present in the insoluble nuclear fraction.

**Figure 2:**
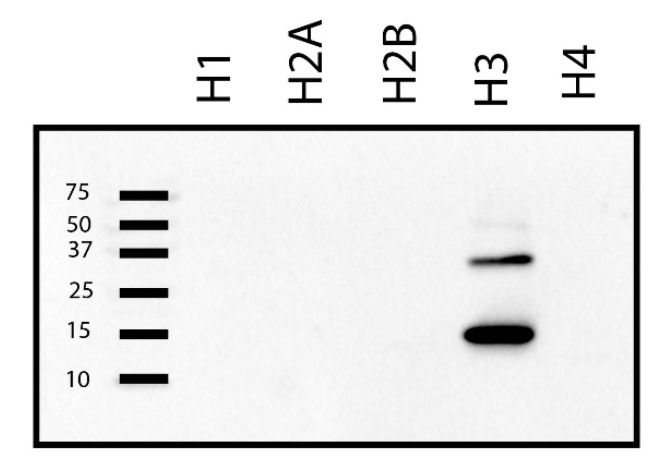
IGFBP-3 binds with histone 3 and no other histone proteins. Equal amount (50 ng) of H1, H2A, H2B, H3 and H4 were separated on 15% gel and far-Western blot analyses was performed using biotinylated IGFBP-3 as indicated in the materials and methods. IGFBP-3 bound to histone 3 and no other histones.

The insoluble nuclear fraction is enriched with nucleosomes that are composed of histone proteins; therefore, we presumed that the ~15 kDa band in the ligand blot (Figure 1A–C) could potentially correspond to one of the histone proteins. Five histone proteins—histone1 (H1; mol. wt.: 21.7 kDa, UniProtKB ID: P07305), histone 2A (H2A; mol. wt.: 13.96 kDa, UniProtKB ID: P0C0S8), histone 2B (H2B; mol. wt.: 13.78 kDa, UniProtKB ID: B4DR52), histone 3 (H3; mol. wt.: 15 kDa, UniProtKB ID: P68431) and histone 4 (H4; mol. wt.: 11 kDa, UniProtKB ID: P62805) are known [34,35]. Of the different histone proteins, H2A, H2B, and H3 have molecular weights that are very close to 15 kDa. To determine which of the histone protein(s) corresponds to the ~15 kDa band in the insoluble fraction, equal amount (50 ng) of purified recombinant H1, H2A, H2B, H3, and H4 were resolved on a 15% SDS-polyacrylamide gel and far-Western blot analysis was performed using biotinylated IGFBP-3. Under the given experimental conditions, our results indicated that on denaturing gel electrophoresis, IGFBP-3 could bind with H3 and not any other histone proteins (Figure 2).

**Figure 3:**
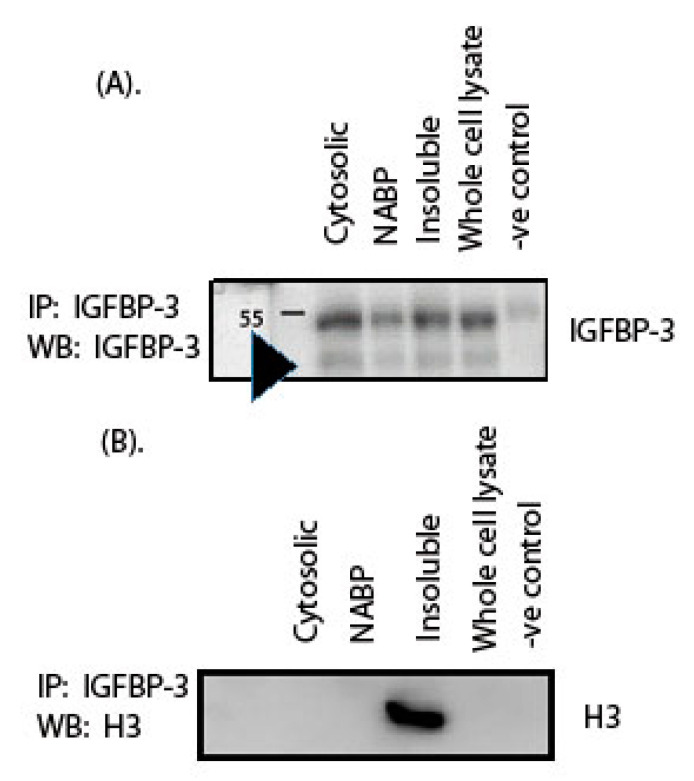
Histone 3 is a nuclear IGFBP-3 binding protein. HepG2 cells were cultured until 80% confluency followed by starvation in serum deprived media overnight. Cells were fractionated into cytosolic and nuclear fractions. The nuclear fraction was further sub-fractionated into nucleic acid binding protein (NABP) fraction and insoluble nuclear fraction. Fractions were crosslinked with EGS followed by preclearing with Sepharose G. IGFBP-3 was immunoprecipitated (IP) either with human IGFBP-3 antibody or rabbit IgG (-ve control) and resolved on a 12% gel followed by transferring onto PVDF membrane. (**A**). The membrane was probed with IGFBP-3 antibody and Western blot (WB) was performed. The arrow represents 40 kDa, corresponding to IGFBP-3. (**B**). The same membrane was stripped and probed with histone 3 (H3) antibody.

We performed co-immunoprecipitation experiments to confirm that the ~15 kDa protein identified by us is indeed an H3 protein. Whole cell lysate, cytosolic, NABP and nuclear insoluble fractions from HepG2 cells were used for the co-immunoprecipitation experiment. The samples were crosslinked with EGS (ethylene glycol bis(succinimidyl succinate) followed by preclearing. IGFBP-3 and its interacting partners were pulled down by IGFBP-3 antibody. The pulled down complex was subjected to Western blot analysis. The immunoblot was first probed for IGFBP-3 to confirm that IGFBP-3 is present in the pulled down co-immunoprecipitated complex. A band at 40 kDa was observed corresponding to IGFBP-3 (Figure 3A). Rabbit IgG was used as a negative control, and there was no band observed at 40 kDa, indicating that the co-immunoprecipitation was specific to IGFBP-3 (Figure 3A). The membrane was then stripped and probed with an H3 antibody. A band at 15 kDa molecular weight was observed corresponding to H3 in the pulldown (Figure 3B) complex. The complete blot showing the interaction between IGFBP-3 and H3 in the co-immunoprecipitation experiment is shown in the Supplementary Section (Figure S2). The co-immunoprecipitation experiment results followed by Western blot analysis indicate that the nuclear IGFBP-3 binds with H3, suggesting that the nuclear IGFBP-3 could associate with the DNA-histone complex.

Histones are a highly conserved group of basic proteins, which are components of chromatin, primarily associated with DNA in vivo. Histones play an essential role in the packaging of DNA, resulting in nucleosome formation, the primary level of DNA packaging. Our results indicate that nuclear IGFBP-3 associates directly with H3 (Figure 3) and not with other histone proteins (Figure 2). However, in the IGFBP-3 pulldown, along with H3 other histone proteins are expected to be present since they are part of nucleosomes.

**Figure 4:**
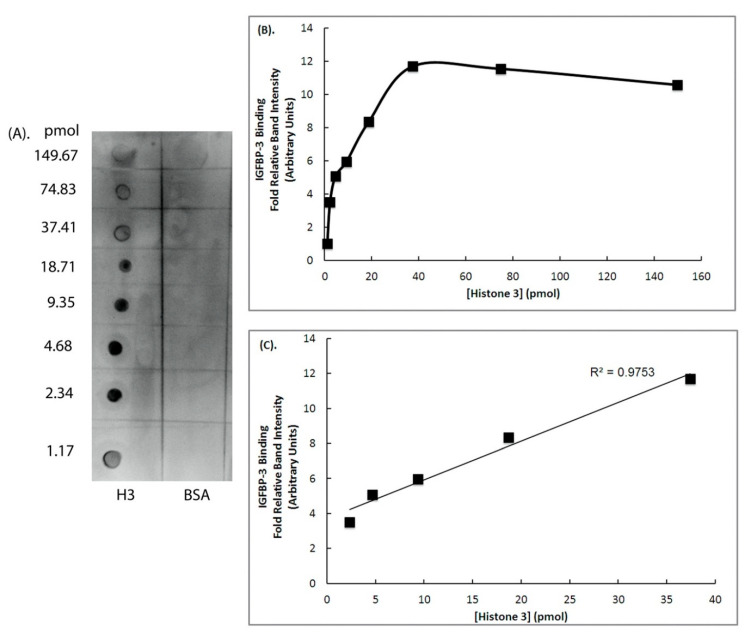
IGFBP-3 is a histone 3 binding protein. Equimole concentrations of recombinant histone 3 protein, and BSA were spotted onto a nitrocellulose membrane followed by probing with biotinylated IGFBP-3. IGFBP-3 ligand dot blot was performed as indicated in the Materials and Methods. (**A**) Image shows the IGFBP-3 ligand dot blot. (**B**) IGFBP-3 binding with histone 3, plot between various concentrations of histone 3 and relative band intensities of the dots obtained, the value corresponding to the lowest concentration was scaled to 1. (**C**). Plot showing a proportional increase in the binding between IGFBP-3 and histone 3. Figure (**C**) shows the enlargement of linear range displayed in figure (**B**).

Once we established that the ~15 kDa protein binding to IGFBP-3 is an H3 protein, we further determined the binding affinity of H3 with IGFBP-3 in vitro. For this, an IGFBP-3 ligand dot-blot assay was performed using varying concentrations of recombinant H3 protein (149.66 pmol–1.17 pmol) that were spotted on a nitrocellulose membrane (Figure 4A). Thereafter, dot blot was incubated with biotinylated IGFBP-3. The IGFBP-3-H3 complexes were detected using biotin-streptavidin-HRP that was developed using chemiluminescence agent (Figure 4A). Equimole concentrations of BSA was used as a negative control (Figure 4A). Dot blot experiment further confirmed that biotinylated IGFBP-3 could bind to H3. The binding affinities between IGFBP-3 and H3 were determined by quantitively analyzing the intensities of the dots. The IGFBP-3 binding to varying concentrations of H3 demonstrated a hyperbolic curve (Figure 4B). There was a linear increase in the binding between IGFBP-3 and H3, following which saturation was reached (Figure 4B). A linear correlation between IGFBP-3 binding to H3 was observed between the concentrations of 0.469 µM (2.33 pmol) and 7.5 µM (37.42 pmol) (Figure 4C).

## 3. Discussion

We have demonstrated that IGFBP-3 protein can bind with H3 protein. Eukaryotic histones proteins are a group of basic proteins that are highly conserved. They are involved in the compaction of DNA forming nucleosomes, which is the first level of the packaging of DNA. Each nucleosome is a hetero-octamer comprising of pairs of H3-H4 dimers associated with pairs of H2A-H2B dimers [36,37,38,39] and 147 bp of DNA is wrapped in 1.7 turns around the octamers of histone proteins arranged as a nucleosome [40].

IGFBP-5 is structurally and functionally similar to IGFBP-3 [41] and has also been reported to bind with H3, which was demonstrated by co-immunoprecipitation and proximity ligation assays [42]. The binding of IGFBP-5 with H3 can regulate the transcription. Studies using deletion mutations of IGFBP-3 from lamprey demonstrated the presence of transactivation domain in the *N*-terminal domain [43]. Furthermore, transcriptional regulatory activity has been shown to be associated with the *N*-terminal domain of human IGFBP-3, IGFBP-4 and IGFBP-5 proteins [42]. Interestingly, transactivation domain was present in the *N*-terminal domain of IGFBP-5 protein, and the IGFBP-5 peptide containing *N*-terminal domain exhibited transactivation, however, the full-length IGFBP-5 protein failed to exhibit transactivation, suggesting the presence of negative regulatory elements within the IGFBP-5 protein [42]. The full-length IGFBP-5 protein could suppress transcription, and it was demonstrated that the transcription repression activity was present in the mid-linker domain and the C-terminal domain [42]. *N*-terminal domain of human IGFBP-3 is also known to possess transactivation domain; however, it is unknown if the full-length protein has transcription negative regulatory elements and, therefore, the effect of the full-length protein on the transactivation of IGFBP-3 remains unknown [42].

Our results indicate that the H3 and IGFBP-3 proteins bind to each other in a concentration-dependent manner and the linear range is in picomoles. Using dot-ligand blot, the linear range for IGFBP-3 binding to IGF-1 was reported to be between 25–1000 nmol, in a study led by Cohen [21]. From our study, it is evident that the binding of IGFBP-3 with H3 does not fall in the range of IGF-1 binding. Our results indicate that the binding affinity of H3 is between 2.33–37.42 pmol, suggesting a stronger binding of IGFBP-3 with H3 compared to IGF-1, as shown in ligand dot blot experiment (Figure 4).

The internalization of IGFBP-3 into the nucleus has been reported to be through the direct interaction with importin-β in a non-classical manner [2]. Nuclear IGFBP-3 can function as a transcription factor or a regulator of transcription either directly or indirectly through the interaction with different nuclear hormone receptors [29]. Nuclear IGFBP-3 has also been demonstrated to regulate the process of DNA repair pathways through non-homologous end joining (NHEJ) [44].

The role of IGFBP-3 as a direct regulator of transcription is supported by the study that demonstrated an interaction of IGFBP-3 with RNA polymerase binding subunit 3 (Rpb3), one of the 13 subunits of RNA polymerase [28].

The role of IGFBP-3 as an indirect regulator of transcription through the interaction with nuclear hormone receptor was demonstrated by showing the direct interaction with retinoid X receptor-α (RXR-α), which was essential for the mediation of embryonic growth, development, and differentiation [29]. Apart from RXR-α, IGFBP-3 protein has also been demonstrated to directly interact with other nuclear hormone receptors, RAR-α, VDR, PPAR-γ and THR thus supporting its role in indirect regulation of transcription [30,31,45,46,47,48].

For the first time, this study identified that H3 protein interacts with nuclear IGFBP-3, which could be suggestive of the role of IGFBP-3 in chromatin remodeling. Chromatin structure is known to undergo compaction and relaxation, which is required to regulate critical cellular processes like DNA replication, transcription, recombination repair, and chromosomal stability. The domains of IGFBP-3 and H3 involved in this interaction are not known and will be investigated in the future. Basic histone proteins, including H3 are known to undergo post-translational modifications like methylation, phosphorylation, acetylation at the *N*-terminal region, which may play a role in interaction with IGFBP-3.

Protein-protein interaction between IGFBP-3 and H3 could prove to be vital in influencing structural changes to the genome. Alteration in gene expression is a prime cause for the onset and progression of various diseases, including cancer and other metabolic disorders. Thus IGFBP-3 interactions with H3 could unfold as an essential mechanism of gene expression in several metabolic diseases.

## 4. Material and Methods

### 4.1. Cell Lines

Human hepatocellular carcinoma, HepG2 cells were procured from American Tissue Culture Cells (ATCC, Manassas, VA, USA). Cells were cultured in Eagle’s minimum essential medium (EMEM) adjusted to pH 7.4 supplemented with FBS (10%), l-glutamine (1%), and glucose (0.3%) and penicillin/streptomycin (1%) in a 100 mm tissue culture dishes. C2C12 cells were cultured in Dulbecco’s modified Eagle’s medium (DMEM) adjusted to pH 7.4 supplemented with FBS (10%) and penicillin streptomycin (1%). Mouse embryonic stem cells were cultured in Glasglow’s MEM (GMEM) adjusted to pH 7.4 supplemented with FBS (10%). All tissue culture cells were maintained in CO_2_ incubator (Sanyo, Markham, Canada) supplied with CO_2_ (5%) and humidified air.

### 4.2. Cell Fractionation and Lysis

ESC, C2C12 and HepG2 cells were grown until 80% confluent, the cells were starved for overnight followed by fractionation using QProteome nuclear kit (Catalog # 37582, Qiagen, Toronto, ON, Canada,) as per the manufacturer’s instructions. The cells were separated into cytosolic and nuclear fractions, the nuclear fraction was further sub-fractionated into nucleic acid binding protein (NABP) nuclear fraction and insoluble nuclear fraction containing nucleosomes.

### 4.3. Whole Cell Lysis

HepG2, C2C12 and mouse ESC cells were cultured until 80% confluency. The cells were starved for 12 h in medium deprived of FBS. The cells were lysed at 4 °C on an ice bath for 10 min in a buffer comprising of HEPES (50 mM; pH 7.4), sucrose (150 mM), sodium orthovanadate (2 mM), β-glycerophosphate (80 mM), sodium fluoride (10 mM), sodium pyrophosphate (10 mM), sodium EGTA (2 mM), sodium EDTA (2 mM), triton X-100 (1%), SDS (0.1%), phenyl methyl sulphonyl fluoride (1 mM), and protease inhibitor cocktail for mammalian cell culture (100 μL). The lysates were transferred into an eppendorf and spun at 4 °C for 3 min at maximum speed, the supernatant was stored at −20 °C until future use and the pellet was discarded.

### 4.4. Ligand Blotting

Ligand blot is an affinity-based technique that utilizes biotin-tagged IGFBP-3 to bind with its interacting proteins that are separated based on molecular weight on SDS-PAGE and transferred on the nitrocellulose membrane. The protein-protein interactions are visualized using biotin-streptavidin-horseradish peroxidase complex with chemiluminescence reagent [24,49,50,51]. Proteins were resolved using a polyacrylamide gel (12%) and transferred onto PVDF membrane. The membrane was probed with biotinylated IGFBP-3 (2.5 μg/10 mL) (Catalog # BP330-G2.5, Eagle Biosciences, Amherst, NH, USA). The excess of reagent was washed with PBST-BSA and incubated using HRP-tagged streptavidin (Catalog N100, Thermo Fisher Scientific, Waltham, MA, USA), the excess of which was washed three times with PBST-BSA for 10 min at room temperature. The blot was developed using chemiluminescence reagent (Clarity™ Western ECL Substrate from Bio-Rad) and was visualized using gel doc (Bio-Rad, Montreal, QC, Canada).

### 4.5. Ligand Dot Blot

Varying concentrations of recombinant full-length human histone 3 (Catalog # SRP0177, Sigma Aldrich, St. Louis, MO, USA) were spotted on nitrocellulose membrane (0.2 μm; Bio-Rad, Montreal, QC, Canada). Equimole concentrations of BSA (Sigma Aldrich, St. Louis, MO, USA) was used as a negative control. The membrane was allowed to dry at room temperature for an hour followed by blocking in BSA (1%) in TBST, for 2 h to block the nonspecific sites. The membrane was probed with biotinylated IGFBP-3 (1 µg/mL) suspended in BSA-TBST overnight at 4 °C. Excess reagents were next washed with TBST three times, 10 min each. The membrane was incubated with HRP-tagged streptavidin secondary antibody at room temperature for an hour. Excess reagents were washed with TBST, three times and developed using chemi-luminescence reagent (Clarity™ Western ECL Substrate, Bio-Rad) and was visualized using gel doc, relative band intensities of the dots were measured using ImageLab (Bio-Rad, Montreal, Canada (version 6.0.1)).

### 4.6. Far-Western Blot Analyses

50 ng of recombinant human H1 (Catalog # H917, Sigma Aldrich, St. Louis, MO, USA), recombinant human H2A (Catalog # H2042, Sigma Aldrich, St. Louis, MO, USA), recombinant human H2B (Catalog # H2167, Sigma Aldrich, St. Louis, MO, USA), recombinant human H3 and recombinant human H4 (Catalog # H2667, Sigma Aldrich, St. Louis, MO, USA) were separated on a 15% gel and was transferred on PVDF membrane and ligand blotting was performed using biotinylated IGFBP-3.

### 4.7. Co-Immunoprecipitation

HepG2 cells were fractionated into cytosolic fraction and nuclear fraction and the nuclear fraction was further sub-fractionated into nucleic acid binding protein (NABP) nuclear fraction and insoluble nuclear fraction containing nucleosomes. Various fractions and whole cell lysate were cross linked using ethylene glycol bis(succinimidyl succinate) (EGS) (Catalog #21565, Thermo Fisher Scientific, Waltham, MA, USA) for 30 min at 4 °C. Tris (50mM, pH 7.5) was used to quench the reaction followed by preclearing with Sepharose G beads. Either human IGFBP-3 antibody (Santa Cruz Biotech, Dallas, TX, USA) or rabbit IgG (Sigma Aldrich) was incubated with various fractions for overnight at 4 °C on a rotator followed by incubation with Sepharose G beads (100 μL) (Bio-Rad). The beads were washed with lysis buffer three times (1 mL) and eluted with Laemmeli buffer (20 μL) comprising of Tris (150 mM; pH 6.8), dithiothreitol (300 mM), SDS (6%), glycerol (30%) for 5 min at room temperature. Supernatant from the elution was transferred into a 1.5 mL microcentrifuge tube and was analyzed using Western blot analysis.

### 4.8. Western Blot Analysis

Western blot analyses was performed according to the method developed by Towbin [52]. Aliquots corresponding to 50 μg protein were mixed with Laemmeli buffer and heated on a heating block (Accublock Digital Dry Bath, MBI Equipment, Pickering, Canada) adjusted to 95 °C for 5 min and spun at 664× g. The samples were resolved on a polyacrylamide gel (12–15%) and were transferred onto a blotting grade polyvinylidene fluoride (PVDF) membrane (Bio-Rad) that was activated with methanol as per manufacturer’s instructions. The membrane was blocked using non-fat dry milk (5%) dissolved in PBS containing tween-20 (0.02%; PBST) for 1h to block the non-specific sites. The membranes were probed with rabbit polyclonal IGFBP-3 antibody (1:1000 dilution, SantaCruz Biotech Dallas, TX, USA,), goat polyclonal histone 3 antibody (SantaCruz Biotech, 1:1000 dilution), rabbit monoclonal tubulin antibody (1:1000 dilution, Thermo Fisher Scientific, Waltham, MA, USA) in 5% milk-PBST, overnight at 4 °C. The membranes were washed three times using PBST for 10 min each, followed by incubation with appropriate secondary antibody for 1 h at room temperature, the excess of reagents were washed with PBST three times for 10 min each. Visualization of hybridization was carried out using chemiluminescence agent Clarity™ Western ECL Substrate (Bio-Rad) and gel doc system (Bio-Rad).

### 4.9. Protein Estimation

Protein estimation was performed using bicinchoninic acid (BCA) Micro BCA^TM^ Protein assay kit (Thermo Fisher Scientific, Waltham, MA, USA) according to manufacturer’s instructions and the absorbances were read using SpectraMax 190 Absorbance Microplate Reader (Molecular Devices, San Jose, CA, USA).

## Data Availability

Not applicable.

## Supplementary Materials

The following are available online at https://www.mdpi.com/1422-0067/22/1/407/s1, Figure S1: Determination of purity of subcellular fractions; Figure S2: Nuclear IGFBP-3 binds with histone 3.
