# Supplementary material for: Insulin-Like Growth Factor Binding Protein-3 Binds to Histone 3
Source: Int J Mol Sci. 2021 Jan 2;22(1):407. doi: 10.3390/ijms22010407 (PMC7796407; doi:10.3390/ijms22010407)
Supplement: Supplementary file 1 [file ijms-22-00407-s001.pdf]

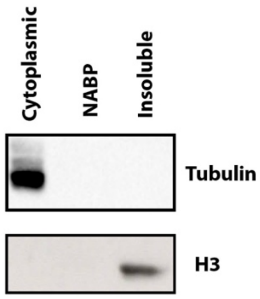

Figure S1: Determination of purity of subcellular fractions. HepG2 cells were cultured until 80% confluent and sub-cellular fractions were isolated as described in the material and methods section. Proteins (20µg) were separated on a 12 % SDS-PAGE and Western blot analysis was performed. The immunoblots were probed for tubulin or histone 3 (H3) antibody. Tubulin and H3 bands are depicted in the figure.

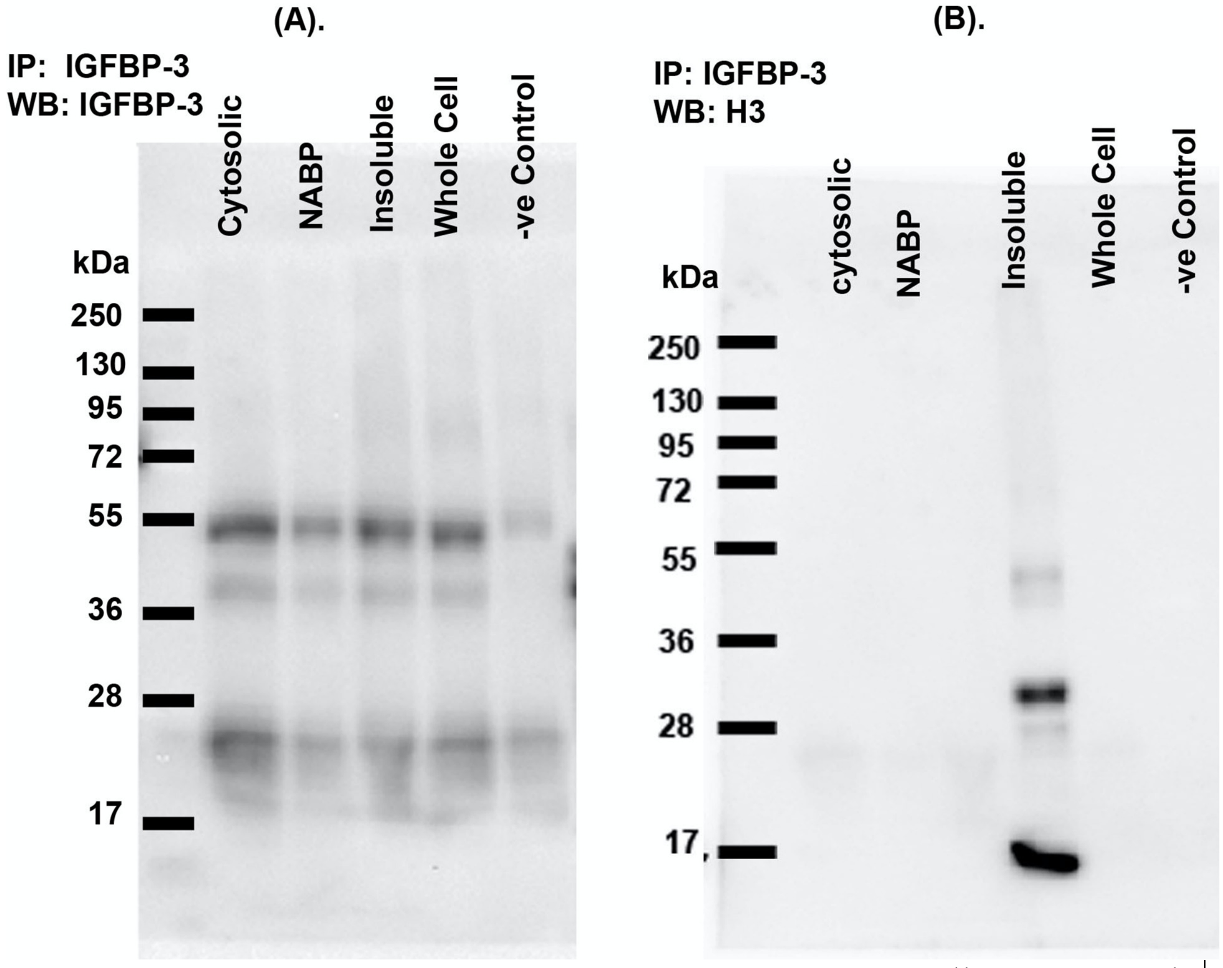

Figure S2: **Nuclear IGFBP-3 binds with histone 3.** HepG2 cells were starved for 12h and were fractionated into cytosolic, nucleic acid binding protein (NABP) nuclear fraction and insoluble nuclear fraction and whole cell lysate. Proteins were crosslinked using EGS and co-immunoprecipitation was performed using IGFBP-3 antibody or rabbit IgG (-ve control) from various fractions and whole cell lysate. The co-immunoprecipitates were resolved on 12% gel and Western blot (WB) analysis was performed using (A). IGFBP-3 antobody and (B). histone 3 (H3) antibody.
